# Effects of Chitosan Oligosaccharides on Human Blood Components

**DOI:** 10.3389/fphar.2018.01412

**Published:** 2018-12-03

**Authors:** Xi Guo, Tong Sun, Rui Zhong, Lu Ma, Chao You, Meng Tian, Hao Li, Chengwei Wang

**Affiliations:** ^1^Department of Neurosurgery, West China Hospital, Sichuan University, Chengdu, China; ^2^Neurosurgery Research Laboratory, West China Hospital, Sichuan University, Chengdu, China; ^3^Institute of Blood Transfusion, Chinese Academy of Medical Sciences & Peking Union Medical College, Chengdu, China; ^4^West China Brain Research Centre, West China Hospital, Sichuan University, Chengdu, China; ^5^Department of Integrated Traditional and Western Medicine, West China Hospital, Sichuan University, Chengdu, China

**Keywords:** chitosan oligosaccharide (COS), red blood cells (RBC), coagulation, complement, platelet

## Abstract

Chitosan oligosaccharide (COS) is known for its unique biological activities such as anti-tumor, anti-inflammatory, anti-oxidant, anti-bacterial activity, biological recognition, and immune enhancing effects, and thus continuous attracting many research interests in drug, food, cosmetics, biomaterials and tissue engineering fields. In comparison to its corresponding polymer, COS has much higher absorption profiles at the intestinal level, which results in permitting its quick access to the blood flow and potential contacting with blood components. However, the effects of COS on blood components remain unclear to date. Herein, two COS with different molecular weight (MW) were characterized by FTIR and ^1^H NMR, and then their effects on human blood components, including red blood cells (RBCs) (hemolysis, deformability, and aggregation), coagulation system [activated partial thromboplastin time (APTT), prothrombin time (PT), thrombin time (TT), and the concentration of fibrinogen (Fib)], complement (C3a and C5a activation), and platelet (activation and aggregation), were comprehensively studied. In the case of RBCs, COS exhibited a low risk of hemolysis in a dose and molecular weight dependent manner and the irreversible aggregation was observed in their high concentration. For coagulation system, COS has a mild anticoagulation activity through blocking the intrinsic coagulation pathway. In addition, COS showed no effect on complement activation in C3a and C5a and on platelet activation while inhibition of platelet aggregation was evident. Finally, the mechanism that effects of COS on blood components was discussed and proposed.

## Introduction

Chitosan oligosaccharide (COS) is an oligomer of chitosan with an average molecular weight (MW) < 5,000 Da, and its chemical structure, like chitosan, is a linear binary copolymer consisting of β-1, 4-linked 2-acetamido-2-deoxy-β-D-glucopyranose (GlcNAc) and 2-amino-2-deoxy-β-D-glucopyranose (GlcN) (Kumar et al., 2004; Muanprasat and Chatsudthipong, 2017). The great research interest on this oligomer results not only from its physical-chemical properties such as better water solubility and cationic nature at neutral pH, but also from its biological activities, e.g., anti-tumor, anti-inflammatory, anti-oxidant, anti-bacterial activity, biological recognition, and immune enhancing effects (Liu et al., 2009; Lu et al., 2014, 2015; Zhang et al., 2014, 2018; Huang et al., 2016; Kunanusornchai et al., 2016; Mattaveewong et al., 2016; Ding et al., 2017; Kalagatur et al., 2018). These unique properties and activities continuously attract many research interests in drug, food, cosmetics, biomaterials and tissue engineering fields (Swiatkiewicz et al., 2015; Lee et al., 2017; Bai et al., 2018; El-Sayed et al., 2018; Nan et al., 2018).

Although the use of COS has been broadened in many research and applied areas, there are some dilemmas that need to be elucidated. For example, it is well-known that oral administration of COS has much higher absorption profiles at the intestinal level than that of its corresponding polymer, chitosan, which results in permitting its quick access to the blood flow and potential contacting with blood components (Chae et al., 2005). This is also applicable to chitosan based tissue-engineering scaffolds, because the degraded fragments that mainly composed of COS would finally enter into the blood circulation. For these reasons, the effect of COS on the blood such as its main components, including red blood cells (RBCs), coagulation, protein adsorption, complement, and platelets, has to be taken into account, since the hemocompatibility of the COS has not been reported to date. In general, chitosan formed a coagulum in contact with whole blood where platelets showed distinct adhesion to its surface within a short time, and the clotting time was reported to reduce by 40% compared to whole blood alone (Rao and Sharma, 1997; Chen et al., 2017). As a result, chitosan is normally used as a hemostatic dressing for wound healing in clinic instead of a blood-contact medical device (Muzzarelli et al., 2005). The hemostatic property was attributed to the polycationic characteristic of chitosan and its non-specific binding to cell membranes resulting from the positively charged amino groups along the molecular chains (Benesch and Tengvall, 2002). In the case of COS, however, some different phenomena were observed. Lin et al. compared the whole blood clotting time of COS and chitosan and found that the former showed no hemostatic effect (Lin and Lin, 2003). Similarly, Fernandes et al. studied the interactions of COS with human RBCs and the results showed that no significant hemolysis was evident and the damage of COS on the RBCs was dependent on the concentration and MW of the used samples (Fernandes et al., 2008). The above reports indicated that COS appears to be better hemocompatiblility than chitosan. Besides the positively charged amino groups, there are numerous hydroxyl groups along the molecular chains of the COS. Previous studies on the effect of functional groups such as hydroxyl, carboxyl, and amino groups on the blood components have suggested that hydroxyl groups resulted in a mild anticoagulation as well as complement activation in spite of no report on COS (Sperling et al., 2005). In this regard, it can be expected that the hydroxyl groups along the molecular chains of COS probably also have some specific effects on the blood components.

**Figure 1:**
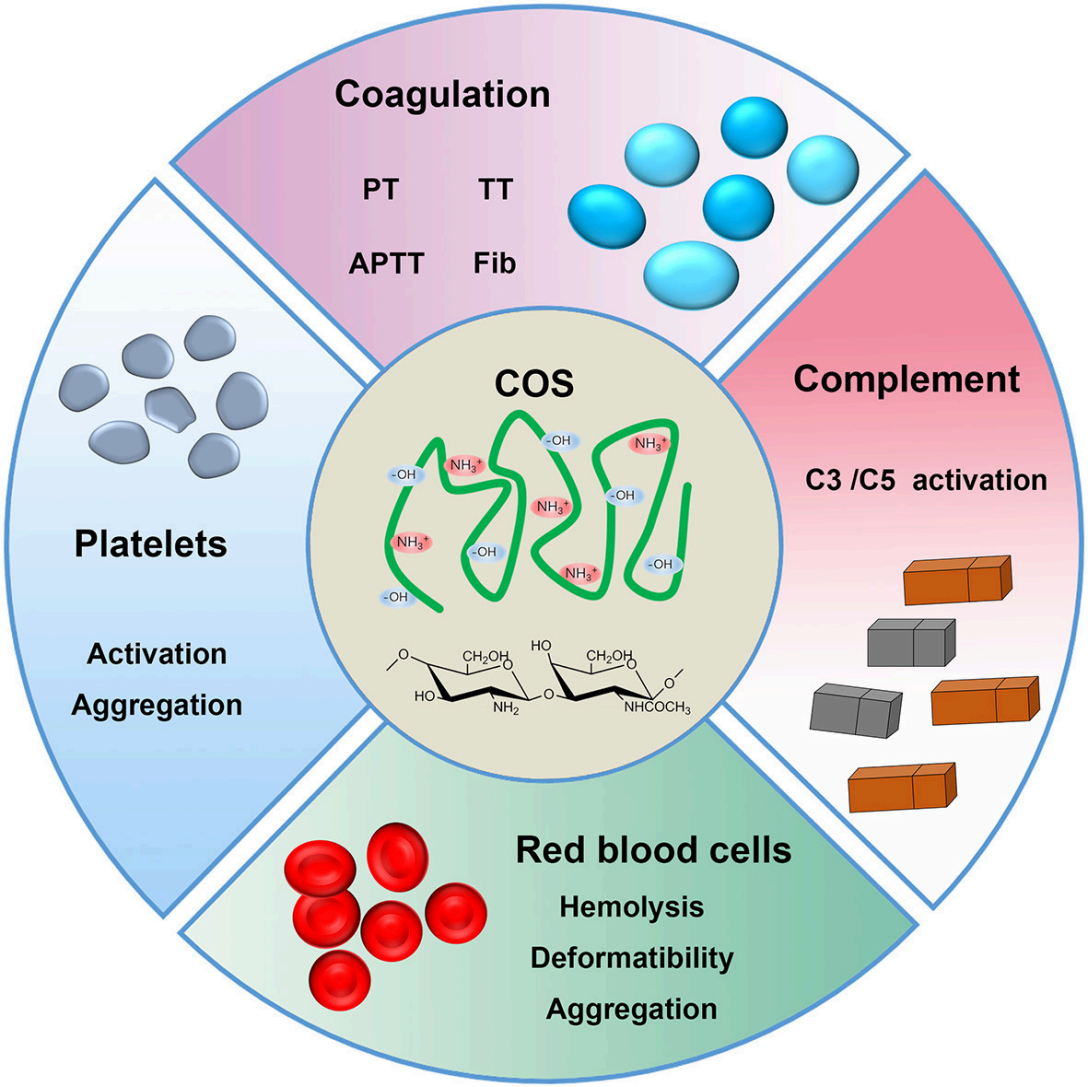
Schematic presentation of COS and blood components.

Herein, we hypothesize that COS would affect the blood components in a different manner comparable to chitosan, which might be related with its MW and functional groups along the molecular chains. To address this hypothesis, the effects of COS with two different MW on the human blood components were comprehensively investigated in this work (Figure 1). Specifically, the effect of COS on RBCs included hemolysis, deformability and aggregation; the blood coagulation was evaluated by coagulation time, including prothrombin time (PT), activated partial thromboplastin time (APTT) and thrombin time (TT), and the concentration of fibrinogen (Fib); for complement system, complement 3a (C3a) and 5a (C5a) using ELISA method were considered; and in terms of platelet, platelet activation, and aggregation were determined. Based on above results, the mechanism of action was discussed and proposed.

## Materials and methods

### Materials preparation

Two COS samples with distinct MW, 3 (COS-3K) and 5 (COS-5K) kDa, were purchased from Zhejiang Aoxing Biotechnology Co., Ltd. (Zhejiang, China). Both samples were obtained by enzymatic hydrolysis of chitosan derived from crab shells. PEI and Heparin sodium were purchased from Aladdin Co., Ltd. (Chengdu, China). Coagulation-related kits were purchased from Union Bio-tech Co., Ltd. (Chengdu, China). The ELISA Kit II was purchased from Becton-Dickinson Co., Ltd, (USA). Flow cytometry-related agents, anti-CD61-fluorecein isothiocyanate (FITC) and anti-CD62p-phycoerythrin (PE) were purchased from BD Pharmingen, BD Bioscience Co., Ltd. Adenosine-diphosphate and epinephrine, which were platelet aggregation-inducers, were purchased from Kelong Co., Ltd. (Chengdu, China). COS-3K/5K were mixed with normal saline to get the COS stock solution.

### Structure characterization

The structure of the two COS was characterized by Fourier transform infrared spectra (FTIR) and ^1^H nuclear magnetic resonance spectroscopy (NMR). FTIR analyses of the COSs were recorded with KBr compressed pellets on a Nicolet 670 FT-IR Spectrometer. ^1^H NMR analyses were recorded on a Bruker AV II-400MHz spectrometer. The DD was calculated by ^1^H NMR according to equation (1), where “A_CH3_” and “A_GlcNH−2_,” respectively correspond to the integral of the N-acetyl proton signal and H-2 proton signal of GlcN units.

(1) $$\begin{array}{l} DD\left(\%\right)=1-\frac{\frac{1}{3}A_{CH_{3}}}{\frac{1}{3}A_{CH_{3}}+A_{GlcNH-2}}\times 100 \end{array}$$

### Blood collection

The study was approved by Ethical Committee of Institute of Blood Transfusion, Chinese Academy of Medical Sciences & Peking Union Medical College. Anticoagulated whole blood samples (3.8% sodium citrate/blood 1:9) and fresh frozen plasma (FFP) anticoagulated with citrate-phosphate-adenine used in this study were obtained from Chengdu Blood Center. For RBC tests, RBC suspension was gained by washing anticoagulated whole blood with normal saline for three times and then adjusting hematocrit (HCT) to 10%. The whole blood samples were centrifuged at 1,200 g for 20 min under 4°C to obtain platelet-poor plasma (PPP) and at 150 g for 10 min under 4°C to acquire platelet-rich plasm (PRP).

### Red blood cells

#### Hemolysis

Washed RBC suspension with 10% HCT (270 μl) was mixed with COS-3K/5K solution (30 μl) to obtain a final COS concentration of 0.01, 0.1, 0.5, 1 mg/ml. The mixtures then were gently centrifuged at 1,500 g for 5 min after 1 h incubation under 37°C. Supernatant (0.02 ml) obtained after centrifuge was added to ortho-tolidine solution (1 ml; 0.2 g in 60 ml acetic acid) and hydrogen peroxide (1 ml; 1 g/L). After incubation for 10 min, the reaction was stopped by mixed with acetic acid (10 ml; 10%). The spectrophotometer was used to measure absorbance at a wavelength of 435 nm. A standard hemoglobin solution with concentration of 100 mg/L was used as a standard control and diluted water (DW) was used as a hemolysis control. To figure out the extent of hemolysis, the following equation was used (Lewis, 1965).

(2) $$\begin{array}{l} Hemolysis\left(\%\right)=\left(\frac{A_{1}}{A_{S}}\times 100\right)\times \frac{100-Hct\left(\%\right)}{C_{Hb}\left(g/L\right)\times 1000} \end{array}$$

*A*_1_ means the absorbance (435 nm) of sample; *A*_*S*_ means the absorbance of standard sample (100 mg/L); Hct (%) means hematocrit; C_Hb_ means concentration of hemoglobin.

#### Deformability

To measure RBC deformability, the washed RBC suspension (270 μl) were incubated with COS stock solution (30 μl) to attain the final concentration of 0.01, 0.1, 0.5, and 1 mg/ml. After 1 h incubation, the suspension was centrifuged at 1,500 g for 4 min and then the precipitate was gently mixed with 1 ml of polyvinylpyrrolidone solution (15% in PBS). A laser-diffraction Ektacytometer system (LBY-BX, Beijing Pencil Instrument Co., Ltd, China) was used. About 0.5 ml of the mixture was added to the system and the sheared between two concentric cylinders of the machine, in which size of gap was 0.5 mm, under four different shear stresses of 0.39, 0.77, 1.54, and 7.7 Pa (corresponding to shear rates of 50, 100, 200, and 1,000) at 37°C. Then the parameter was presented as elongation index (EI) by the system through measuring the diffraction of passing laser. Normal saline and diluted water mixed with RBC suspension were used as standard and positive control, respectively.

#### Aggregation

To determine whether COS can lead to RBC aggregation, anti-coagulated whole blood samples (270 μl) was mixed with COS stock solution (30 μl) to attain the final concentration of 1 and 5 mg/ml for 1 h incubation. The mixtures then were centrifuged at 1,000 g for 3 min. The precipitate (3 μl) and supernatant (40 μl) were gently mixed and mixtures (4 μl) were made into slides. Images of slides were captured by a digital microscope camera. Polyetherimide (PEI), which can lead to obvious erythrocytes aggregation, was used as positive control and normal saline was used as standard control.

#### Coagulation

Activated partial thrombin time (APTT), prothrombin time (PT), thrombin time (TT) and the concentration of fibrinogen (Fib) were measured to determine the effect of COS on coagulation system by a modified strategy according to previous study (Nikitina et al., 2018). Briefly, COS stock solution (60 μl) were mixed with FFP (540 μl) to attain a final concentration of 0.01, 0.1, and 0.5 mg/ml and then was incubated at 37°C for 3 min. A coagulation analyzer (Instrumentation Laboratory ACL ELITE, USA) was used to test the incubated FFP. The parameter range available for the machine is 6 s to 245 s for APTT, 5 s to 165 s for PT, and 3 s to 169 s for TT. NS was used as standard control and heparin (HP, final concentration of 0.75 IU/ml) was used as positive control. Values of above coagulation tests were mean from three measurements and results were expressed as mean ± standard deviation (SD).

#### Complement

Complement activation reflects the effect of COS on complement system. We focused on the activation of human complement C3 and C5 in circulation and measured their cleft fragment C3a and C5a. The method of measurement was followed by the standard protocol of ELISA Kit II (Becton-Dickinson Co., Ltd, USA). Briefly, serum (90 μl) was incubated with COS (final concentration of 0.1 and 1 mg/ml) at 37°C for 1 h. Then incubated serum (100 μl) and standard solution (100 μl) of the kit were, respectively, mixed with ELISA dilution (50 μl) in antibody coated well-sealed under room temperature for 2 h. After that, wells were washed and added detection antibody and enzyme concentrate of the kit for 1-h incubation. Finally, the wells were read and absorbance of 450 nm was measured by spectrophotometer (EON, Bio-Tech CO., Ltd, USA). The concentration of C3a and C5a was calculated according to the standard curve of standard samples of kit.

### Platelet

#### Activation

CD61, a specific marker of platelet surface, and CD62p, the marker of activated platelet, were involved in this test. Platelet-rich plasm (PRP) (90 μl) was incubated with COS stock solution (10 μl) at 37°C for 1 h to obtain a final concentration of 0.1 and 0.5 mg/ml. Incubated PRP (5 μl) was mixed with anti-CD61-fluorecein isothiocyanate (5 μl), anti-CD62p-phycoerythrin (5 μl), and PBS buffer (10 mM, 40 μl) in dark for 15 min and then added 400 μl of PBS buffer. The flow cytometry (Becton-Dickinson, San Jose', CA, USA) were used to measure the number of CD62p-expressing platelets. Normal saline and thrombin (10 U/ml) were used as normal control and positive control, respectively (Zhong et al., 2013).

#### Aggregation

To investigate whether platelet aggregation was affected by COS, PRP (270 μl) was incubated with COS stock solution (30 μl) at 37°C for 1 h. After incubation, we added adenosine diphosphate (0.1 mM, 12.5 μl) and epinephrine (0.15 mM, 12.5 μl) to the incubated PRP to induce platelet aggregation. And the aggregometry (MODEL700, CHRONO-LOG CO. LTD, USA) was used to measure extent of aggregation. PRP incubated with normal saline and thrombin (10 U/ml) were used as control groups.

### Statistical analysis

All results were described as means ± standard deviation (SD). Statistical analysis of all data was performed using SPSS 19 (IBM, Statistic Package for Social Science). A value of *p* < 0.05 was considered as being statistically significant.

## Results and discussions

### Structure characterization of COS

**Figure 2:**
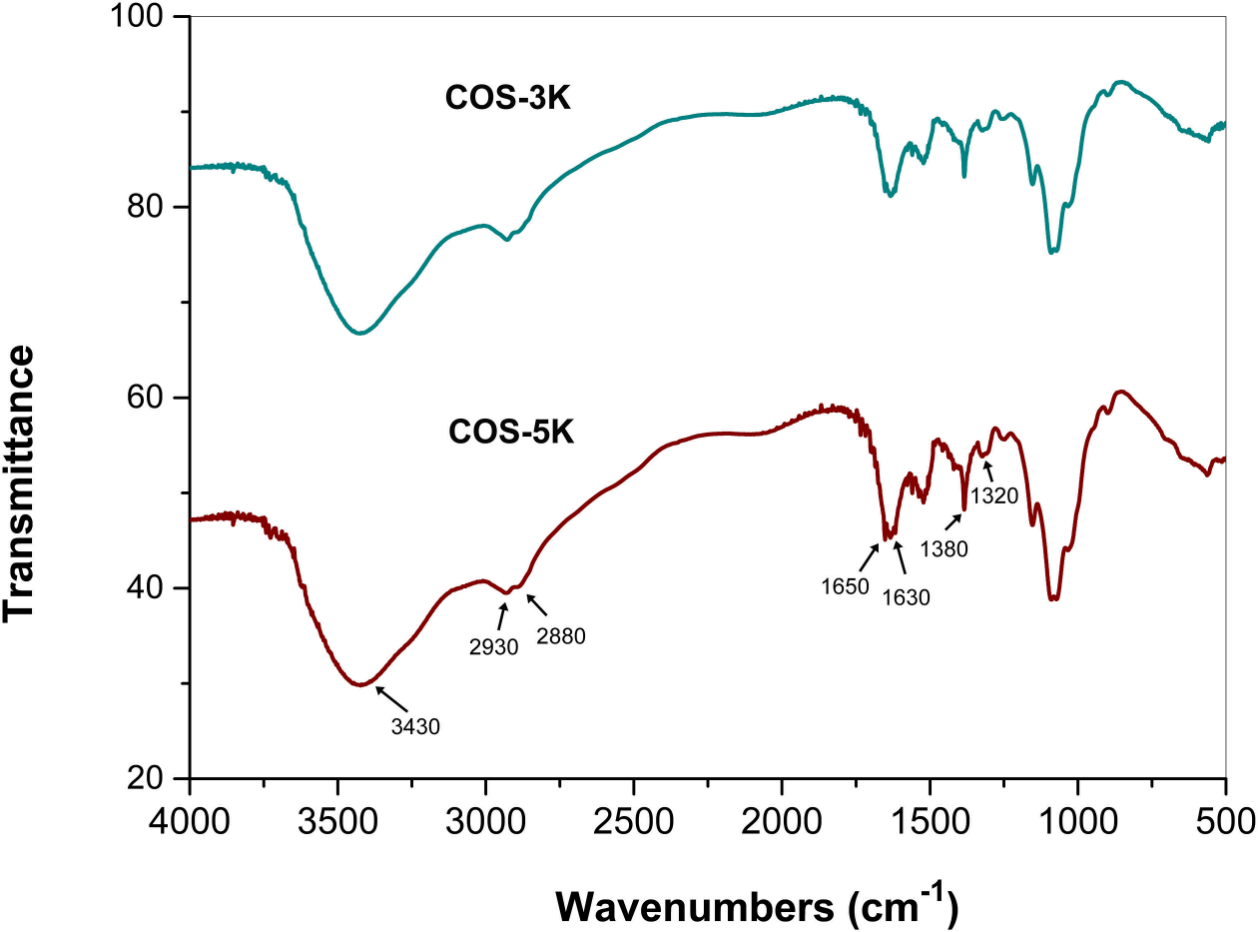
FTIR spectra of two COS.

The FTIR spectra of the two COS are shown in Figure 2, both of which displayed characteristic absorptions that are similar to that of chitosans with high DD as reported previously. The absorption band at around 3,430 cm^−1^ is attributed to the N-H and O-H stretching vibrations (Chen et al., 2016; Wu et al., 2017). The peaks at 2,880 and 2,930 cm^−1^ are assigned to C-H stretching vibrations, and the peaks at 1,650, 1,630, and 1,320 cm^−1^ are, respectively, assigned to amide I, II, and III. The band at 1,380 is corresponds to C-H bending and C-H stretching vibrations, and the band in the range 1,150–890 are assigned to the characteristics of its polysaccharide structure. As previous literature reported, the COS prepared by chemical degradation approaches such as oxidative degradation with peroxide hydrogen shown a weakened amide Iband due to the H-abstraction at C-1 and C-2 during degradation that both led to the partly deamination. However, the two COS samples in this study obtained by enzymatic hydrolysis remain exhibited a strong amide Iband, which indicated that the two COS samples are both degradation products with high DD.

**Figure 3:**
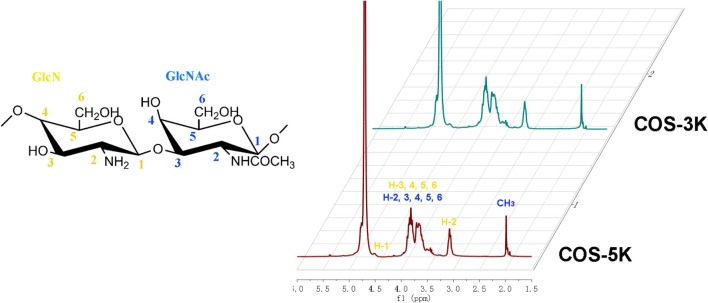
^1^H NMR spectra of two COS.

The chemical structure of the two COS samples was further studied by ^1^H NMR. As shown in Figure 3, the two samples exhibited similar ^1^H NMR spectrum. According to previous literatures, signals at 2.0 and 3.1 ppm are assigned to protons of CH3 and H-2 of GlcN, respectively. In the low field, the signals at 3.5–4.0 ppm are assigned to H-3, 4, 5, 6 of GlcN and H-2, 3, 4, 5, 6 of GlcNAc, and the signals at 4.5 ppm are attributed to H-1 of GlcN (Trombotto et al., 2008). Compared to ^1^H NMR spectrum of the chitosan as previous reported, there is no significant difference for the two samples. In addition, the DD values for the two samples were calculated by ^1^H NMR spectrum according to equation (1). The calculated DD were 88.4 and 87.4% for COS-5K and COS-3K, respectively, which is consistent with the results of the FTIR.

### Red blood cells

**Figure 4:**
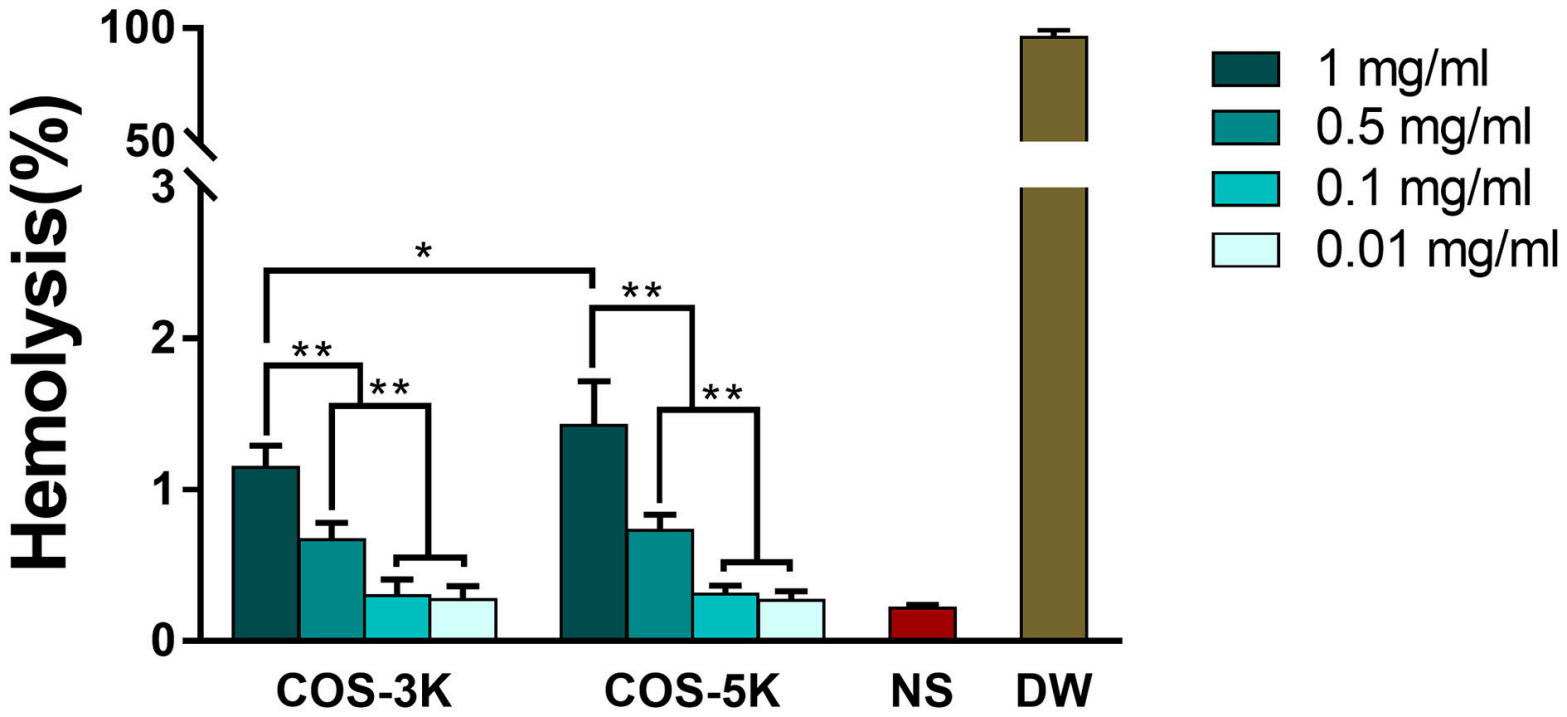
Hemolysis percentage of RBC suspension induced by COS. NS, normal saline; DW, diluted water. ^*^p < 0.05; ^**^p < 0.01.

In order to investigate the effect of two COS on RBCs, three main parameters of RBCs including hemolysis, deformability and aggregation were studied. Hemolysis is a crucial and direct parameter evaluating the safety of biomaterials and defined as the percentage of RBCs lysis when the biomaterial interacting with RBCs suspension (Fernandes et al., 2008). According to ISO10993-5, percentage of hemolysis induced by exogenous materials under 5% was considered as a low risk (Weber et al., 2018). As shown in Figure 4, percentages of hemolysis presented in a dose-dependent manner. As the concentration increasing from 0.1 to 1 mg/ml, the hemolysis increased from 0.30 ± 0.10 to 1.15 ± 0.14% (*p* < 0.01) in COS-3K group and from 0.30 ± 0.06 to 1.42 ± 0.30% (*p* < 0.01) in COS-5K group. When the concentration below 0.1 mg/ml, the hemolysis in both COS groups had no significant difference with that in NS group. We also observed that hemolysis in COS-5K group was significantly higher than that in COS-3K group when the concentration was above 0.1 mg/ml, indicating that the hemolysis of COS also presented as a MW-dependent manner.

**Figure 5:**
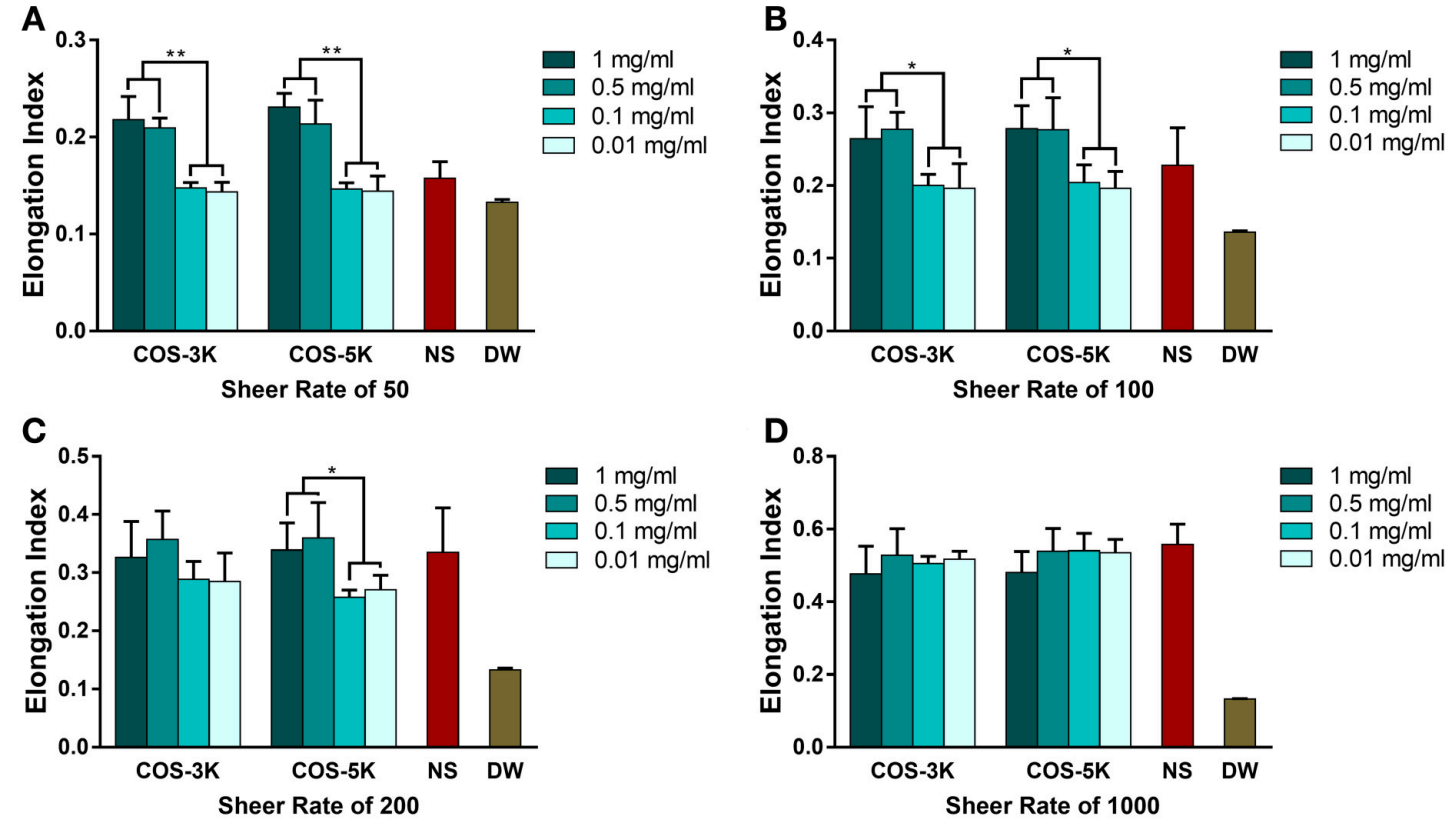
Deformability of RBC detected by a laser-diffraction Ektacytometer system under shear rate of 50 **(A)**, 100 **(B)**, 200 **(C)**, 1,000 **(D)** with the presence of COS with different concentration. NS, normal saline; DW, diluted water. ^*^p < 0.05; ^**^p < 0.01.

As for deformability of RBCs, an index representing the ability of RBCs to change their biconcave disk shape against the exogenous force from vascular walls, we used four different sheer forces in the tests (Zhao et al., 2010). As shown in Figures 5A–D, as sheer force increasing from 0.39 to 7.7 Pa (corresponding sheer rate 50 to 1,000), elongation index was generally increased except in DW group with a stable and lowest EI due to containing no intact RBCs. Under sheer rate of 50 and 100, EI presented a dose dependent manner where EI was significant higher at 0.5 and 1 mg/ml than 0.01 and 0.1 mg/ml. Though under sheer rate of 200, there was no statistical difference on EI between 0.5, 1, and 0.01, 0.1 mg/ml in COS-3K group, EI in COS-5K group had the same trend as under 50 and 100 sheer rate.

**Figure 6:**
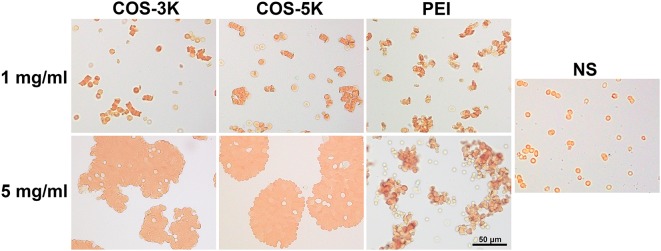
Pictures of RBC aggregation induced by COS under an optical microscope. RBC incubated with PEI was positive control. PEI, polyetherimide; NS, normal saline.

Results of erythrocytes aggregation was in accordance with hemolysis. As shown in Figure 6, at the concentration of 1 mg/ml, RBCs displayed biconcave shape and some erythrocytes rouleaux defined as reversible aggregation were observed compared to normal distribution of RBCs in NS group. However, with the concentration increasing to 5 mg/ml, RBCs all aggregated forming erythrocytes clusters which are irreversible aggregation and the normal shape of erythrocytes was absent. As previous report, amidocyanogen of chitosan could neutralize the negatively charged neuraminic acid residues located on the surface of RBCs (Fernandes et al., 2008). Likely, the positive charge of COS can change the distribution of charge and break the electrokinetic balance on erythrocyte surface leading to aggregation in a dose-dependent manner.

### Coagulation

**Figure 7:**
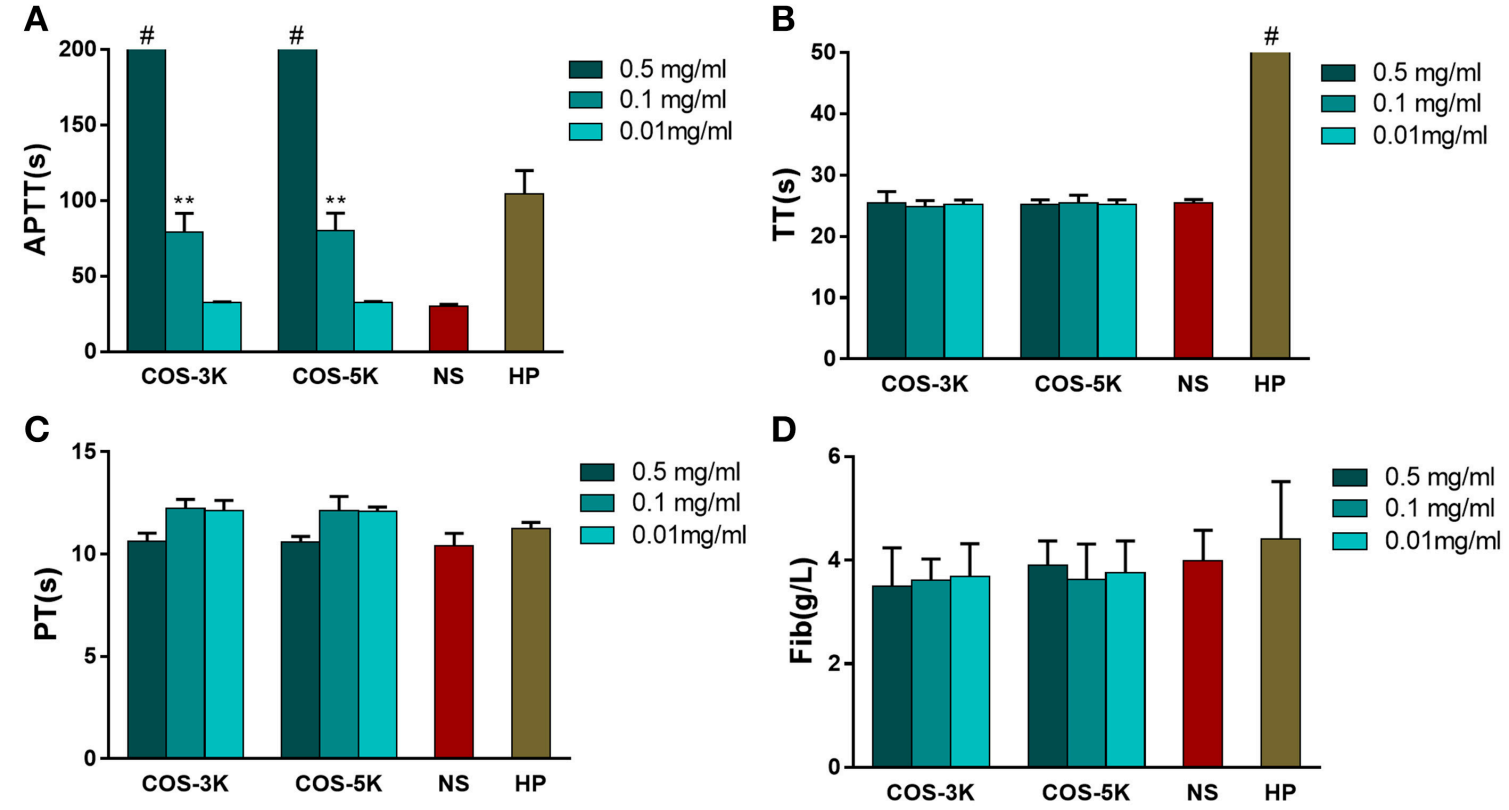
Analysis of influence on coagulation caused by COSs. **(A)** APTT of COSs and heparin. **(B)** TT of COSs and heparin. **(C)** PT of COSs and heparin. **(D)** Concentration of Fib. ^**^p < 0.01 vs. NS group; # means the parameter exceeded machine available. NS, normal saline; HP, heparin.

Effect on coagulation is an essential property of biomaterial due to both anti-coagulation and pro-coagulation can cause fetal complications or side effects on human such as bleeding and thrombosis formation (Zhang et al., 2013). In order to study effect on coagulation, APTT, PT, TT, and Fib were involved in the tests. It is defined as disordered hemostatic property of human blood when APTT changes more than 10 s or PT/TT changes more than 3 s (Yang et al., 2008). In our study, as shown in Figure 7A, high concentration (0.5 and 0.1 mg/ml) COS prolonged APTT compared to NS group (*p* < 0.01) in a dose-dependent manner and the maximum APTT even exceeded the machine available value (>245 s). In contrast, TT was normal in all COS group (Figure 7B). For PT showing in Figure 7C, though it was slightly longer at 0.1 and 0.5 mg/ml group, all the PT values were in normal range when considering 3 s mobility scale. Similar to TT, concentration of Fib in COS groups was equal to that in NS group (Figure 7D). Within our expectation, heparin (0.75 IU/ml) group (TT positive control) showed a prolonged TT value which exceeded 169 s.

APTT and PT, respectively, reflect the status of intrinsic and extrinsic blood coagulation, while TT is used to check the effect on the conversion from fibrinogen to insoluble fibrin induced by thrombin or whether there exist an anticoagulant when APTT and PT are prolonged. Our study indicated COS influenced intrinsic coagulation pathway due to prolonging APTT while PT, TT, and Fib were in normal range. To the best of our knowledge, it is the first time to report that COS has anticoagulation activity through blocking the intrinsic coagulation pathway, which is different from chitosan with pro-coagulant activity. On the other hand, it should be noted that the anticoagulation activity of the COS is mild compared to other anticoagulants such as alginate sulfates, whose APTT was prolonged by 180 s in the concentration of 80 μg/ml, while the APTT of the COS was around 80 s in the concentration of 0.1 mg/ml.

### Complement

**Figure 8:**
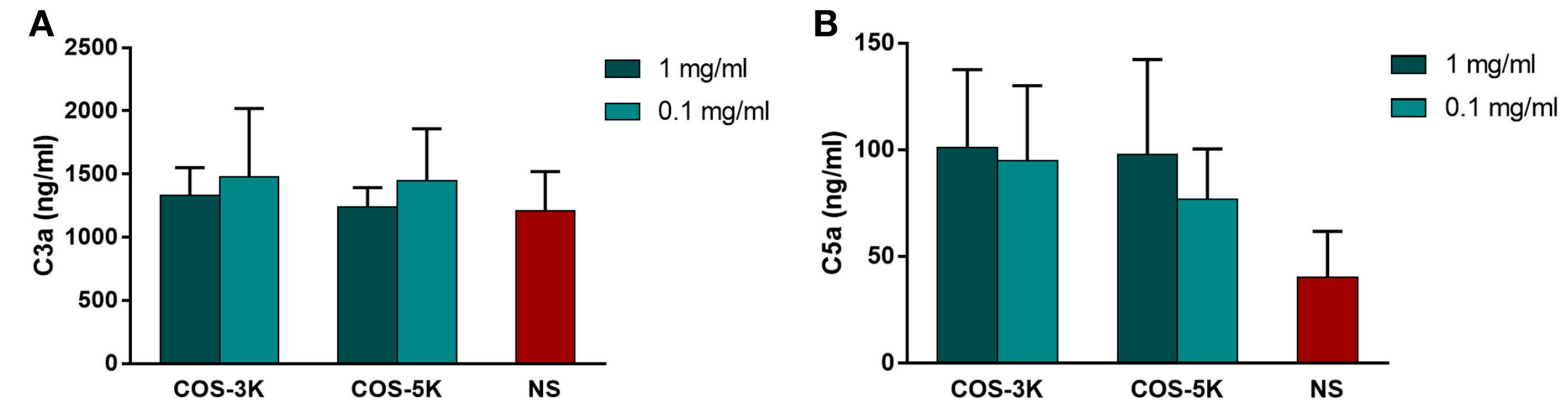
Effects of COS on complement system. **(A)** Concentration of C3a in serum. **(B)** Concentration of C5a in serum. NS, normal saline.

Complement activation is an important indicator of hemocompatibility of biomaterials. Complement system is an innate part of immune system and can be activated via three pathways, classical pathway, alternative pathway and lectin pathway (Coulthard and Woodruff, 2015). Once complement system activated, C3 cleaves to fragments C3a and C3b, and leads to its downstream generation of C5a. As shown in Figure 8, at low concentration, COS seemed to induce more C3a than NS group than that at high concentration. However, the difference has no statistical significance.

Chitosan is suggested to activated complement system by deplete or absorb C3 and C5 proteins from serum (Benesch and Tengvall, 2002). Interestingly, in our study, COS demonstrated relative complement compatibility that C3a and C5a in all groups was in a normal range. Similar to our findings, Marchand et al. reported that chitosan with 80% and 95% DD do not activate complement system for that C3 and C5 bind to chitosan as intact proteins (Marchand et al., 2010). Hence, DD might be a significant factor that determined whether the chitosan induced complement activation.

### Platelet

**Figure 9:**
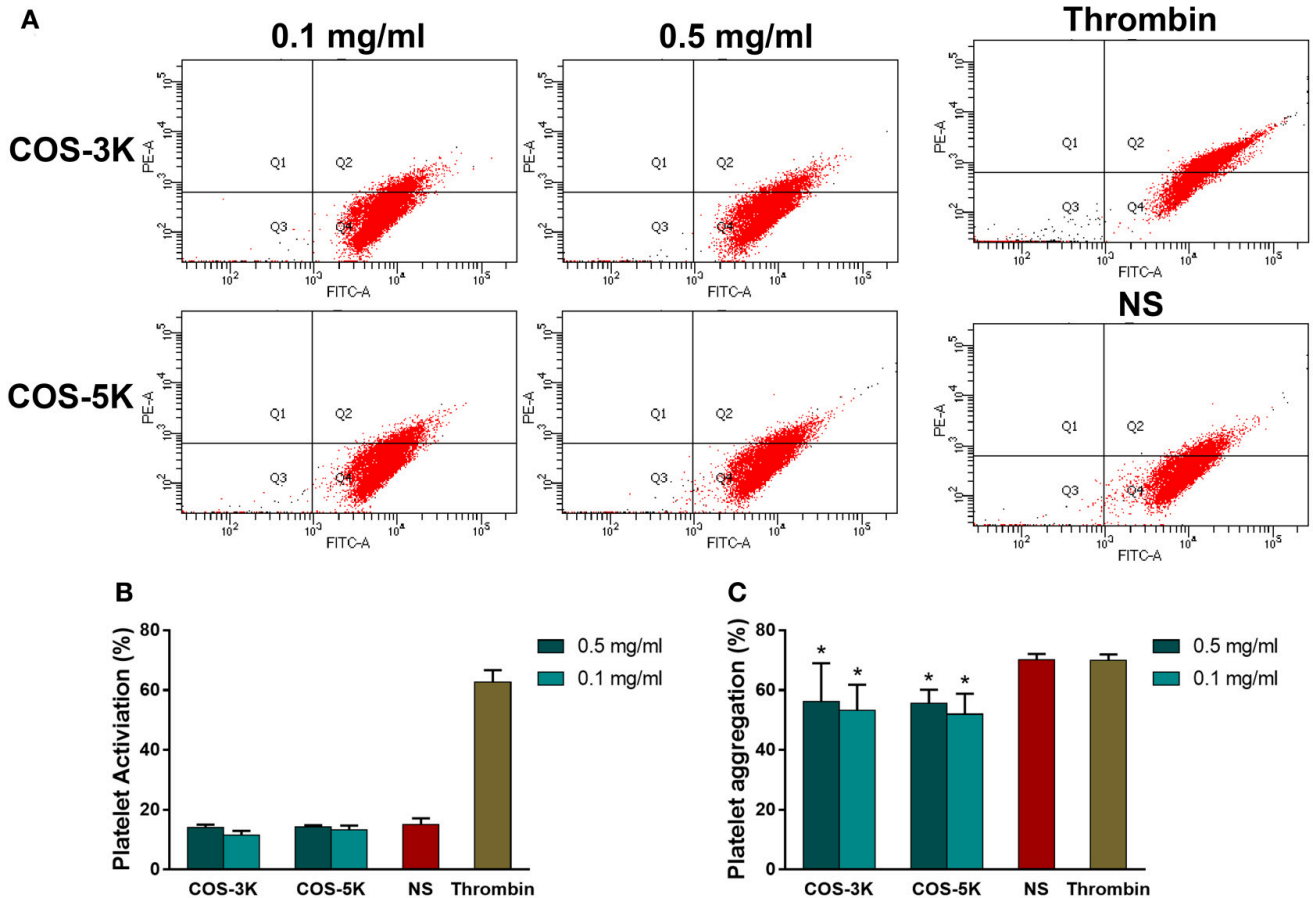
Analysis of influence on platelet caused by COSs: **(A)** Flow cytometry of activated platelets. **(B)** Percentage of platelet activation. **(C)** Effects on Platelet aggregation. NS, normal saline. ^*^p < 0.05.

CD62P, a platelet activation marker, was used to investigate the effect of COS on platelet. As shown in Figures 9A,B, with presence of COS at 0.1 and 0.5 mg/ml, expression of CD62P was in normal range (*p* > 0.05) indicating platelet was not activated by COS. Heparin demonstrated a strong ability to stimulate platelet that CD62P was remarkable high in heparin group. In terms of platelet aggregation, reduced aggregation was observed in all COS groups (*p* < 0.05 for all COS) compared to normal saline (Figure 9C). Moreover, there was no statistical significant difference between two concentrations or two COSs.

Platelet was a vital component in blood participating in coagulation cascade and provided large scale of coagulation factors, such as platelet factor 3 and factor Va, when activated leading to generate platelet micro particles (Gorbet and Sefton, 2004). In coagulation process, induced platelet micro particles promote platelet adherence to fibrin resulting in congregation of platelet and anti-bleeding function. In accordance to coagulation results, COS did not activate platelet and inhibited platelet aggregation indicating it played as an anti-coagulant. Under such concentrations of 0.5 and 0.1 mg/ml, COS with two MW has no difference in ability of inhibiting platelet.

## Discussion of mechanism

**Figure 10:**
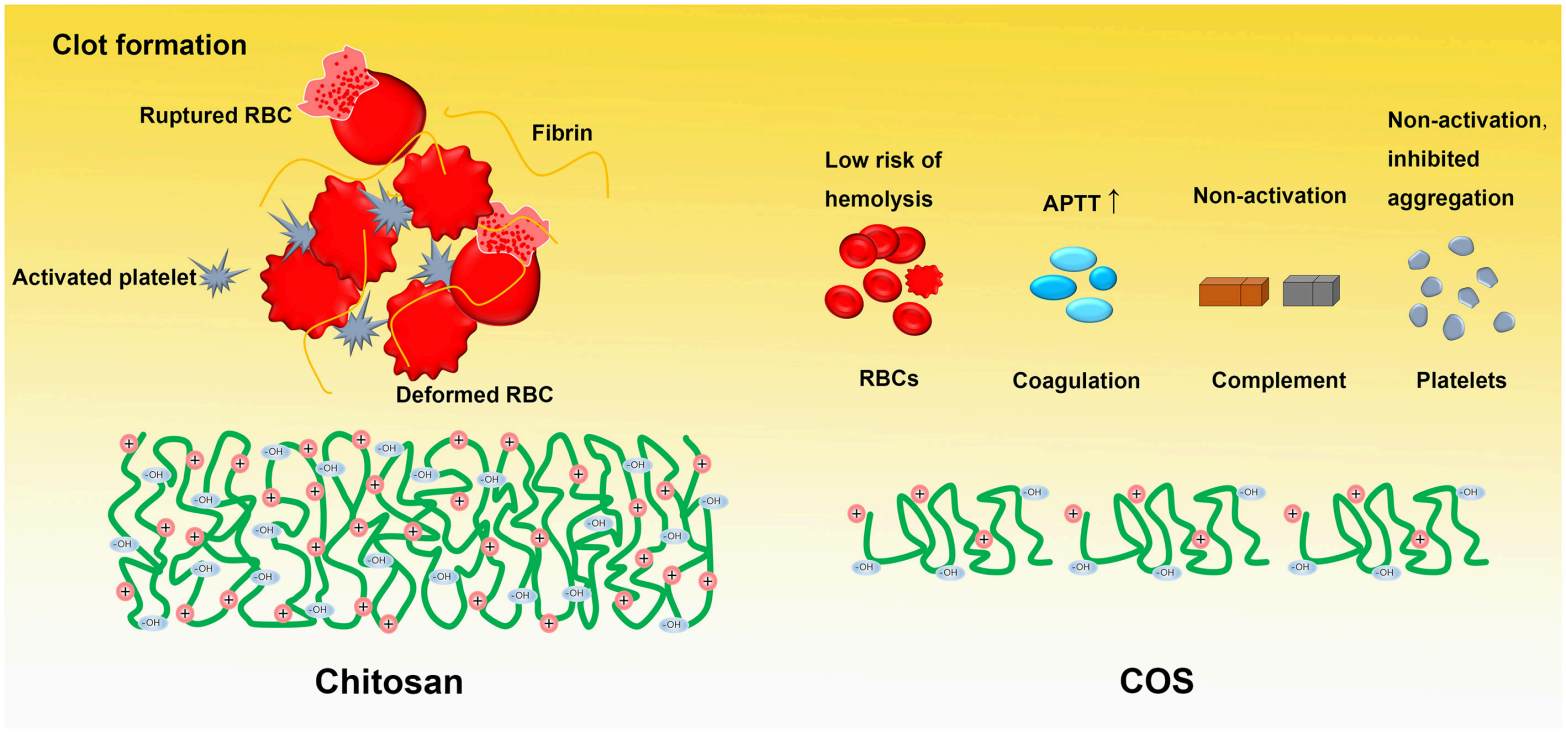
Schematic presentation of the effects of chitosan and COS on blood components.

Chitosan derived from chitin is a cationic polysaccharide that is widely used as a hemostasis, wound dressing, or anti-bacterial material in clinic (Kumar et al., 2004; Muzzarelli et al., 2005). However, application in blood-contacting is occluded by its inferior compatibility with blood components such as procoagulant function and complement activation. Interesting, here it was found that COS exhibited quite different effects on human blood components comparable to chitosan (Figure 10). To explain this phenomenon, a mechanism was proposed as follows based on literatures and our results.

The procoagulant function of chitosan is due to its cationic nature that induced by positively charged amino groups, in which these groups induction of fibrinogen adsorption, activation of platelets, releasing of coagulation factors, and then trigger extrinsic coagulation pathway (Benesch and Tengvall, 2002; Zhang et al., 2013). However, when chitosan was degraded into COS, the decreased MW resulted in decreasing of local positive charge density and thus weakened the electrostatistic interactions between the positively charged amino groups and the blood components in comparison to that of chitosan. In the case of RBCs, the electrostatic interactions results in membrane damage, hemolysis as well as aggregation, while low risk of hemolysis, deformability and aggragation were observed for COS-3k and 5k resulting from the decreased MW and positive charge density. Similarly, the decreased positive charge density may reduce the procoagulant function of chitosan as well. However, our results shown that the both two COS exhibited a mild anticoagulation activity in a dose-dependent manner. The anticoagulation activity probably related with the hydroxyl groups along the molecular chains, since it has been demonstrated that hydroxyl groups are capable of anticoagulation as previously reported (Sperling et al., 2005).

## Conclusion

In summary, the molecular structure of the two COS with different MW was characterized by FTIR and ^1^H NMR, and then the effects of the COS on the human blood components, including RBCs, coagulation system, complement, and platelet, were studied in this work. The results indicated that: (i) COS exhibited a low risk of hemolysis in a dose and MW dependent manner and the irreversible aggregation was observed in their high concentration; (ii) COS has a mild anticoagulation activity through blocking the intrinsic coagulation pathway; (iii) COS has no effect on complement activation in C3a and C5a; (iv) COS has no effect on platelet activation while inhibition of platelet aggregation was evident. Finally, the mechanism of action was discussed and proposed.

## Author contributions

XG: results discussion, writing the article, and hemocompatibility evaluation. TS: results discussion, hemocompatibility evaluation. RZ: hemocompatibility evaluation, statistic analysis. LM: results discussion. CY: evaluating the results of study. MT: article design, NMR spectroscopy, and FTIR characterization. HL and CW: results discussion, statistic analysis.

### Conflict of interest statement

The authors declare that the research was conducted in the absence of any commercial or financial relationships that could be construed as a potential conflict of interest.
